# Who in Europe Works beyond the State Pension Age and under which Conditions? Results from SHARE

**DOI:** 10.1007/s12062-016-9160-4

**Published:** 2016-09-23

**Authors:** Morten Wahrendorf, Bola Akinwale, Rebecca Landy, Katey Matthews, David Blane

**Affiliations:** 1 Institute for Medical Sociology, Centre for Health and Society, Medical Faculty, University of Düsseldorf, Düsseldorf, Germany; 2 International Centre for Life Course Studies in Society and Health, Department of Primary Care and Public Health, Imperial College London, London, UK; 3 Centre for Cancer Prevention, Wolfson Institute of Preventive Medicine, Queen Mary University of London, London, UK; 4 Cathie Marsh Institute for Social Research, University of Manchester, Manchester, UK; 5 International Centre for Life Course Studies in Society and Health, Department of Epidemiology and Public Health, University College London, London, UK

**Keywords:** Extended working life, Working conditions, Share

## Abstract

There is much research about those who exit the labour market prematurely, however, comparatively little is known about people working longer and about their employment and working conditions. In this paper, we describe the employment and working conditions of men and women working between 65 and 80 years, and compare them with previous conditions of those retired in the same age group. Analyses are based on wave 4 data from the Survey of Health, Ageing and Retirement in Europe (SHARE) with information collected between 2009 and 2011 from 17,625 older men and women across 16 European countries. Besides socio-demographic and health-related factors (physical and mental health), the focus lies on employment conditions (e.g. employment status, occupational position and working hours) and on stressful working conditions, measured in terms of low control at work and effort-reward imbalance. In case of retired people, information on working conditions refer to the last job before retirement. Following descriptive analyses, we then conduct multivariable analyses and investigate how working conditions and poor health are related to labour market participation (i.e. random intercept models accounting for country affiliation and adjusted for potential confounders). Results illustrate that people working between the ages of 65 and 80 are more likely to be self-employed (either with or without employees) and work in advantaged occupational positions. Furthermore, findings reveal that psychosocial working conditions are generally better than the conditions retired respondents had in their last job. Finally, in contrast to those who work, health tends to be worse among retired people. In conclusion, findings deliver empirical evidence that paid employment beyond age 65 is more common among self-employed workers throughout Europe, in advantaged occupations and under-favourable psychosocial circumstances, and that this group of workers are in considerably good mental and physical health. This highlights that policies aimed at increasing the state pension age beyond the age of 65 years put pressure on specific disadvantaged groups of men and women.

**Electronic supplementary material:**

The online version of this article (doi:10.1007/s12062-016-9160-4) contains supplementary material, which is available to authorized users.

## Introduction

In response to demographic ageing and its consequences, European governments aim to increase labour market participation of older workers and have started to expand the state pension age beyond age 65. In this context, research has established solid evidence on determinants of employment participation in older ages, including contextual factors (e.g. tax incentives) (Börsch-Supan et al. 2009; Gruber and Wise 1999; Wise 2010) and a number of individual characteristics, such as work and employment conditions. Results of this research are instrumental, as they help to identify aspects that predict retirement behaviour, and thus, help to develop measures to promote extended working lives. However, most studies are based on occupational cohorts, often recruited from specific occupational groups during midlife. In these studies, baseline factors of working populations are usually linked to the likelihood of retirement during a follow-up period. This has an important drawback for existing knowledge, and in particular, about the work and employment conditions of people working beyond the state pension age. Specifically, because information on employment and working conditions refers to midlife, less is known about the working conditions people actually have at work beyond the state pension age. A person’s decision to work beyond the state pension age may, however, also be related to his or her conditions at work beyond state pension age. In other words, much research exists on those aspects in midlife that are prospectively linked with premature exits from the labour market, but comparatively little is known about people working longer, and about their working and employment conditions.

Using wave 4 data from the Survey of Health, Ageing and Retirement in Europe (SHARE), the present paper, describes employment and working conditions of employed men and women aged 65 or older. In addition, we compare conditions of older workers with those which retired people in the same age group had in their last job. Besides socio-demographic factors (e.g. partnership situation, material circumstances) and health (both physical and mental), the focus is on employment conditions (e.g. self-employment, occupational position) as well as psychosocial stress at work. The latter will be measured in terms of two theoretical models of work stress, the demand-control model (Karasek and Theorell 1990) and the effort-reward imbalance model (Siegrist et al. 2004) (see Methods for details). But before presenting our analyses, the next section will briefly summarise how the above mentioned factors (sociodemographic factors, health and working conditions) have previously been related to labour market participation in older age (for a detailled review see: Hasselhorn and Wenke 2015).

Sociodemographic characteristics include sex, partnership situation, parenthood, educational qualifications and economic circumstances. For example, women or people in a partnership seem more likely to have shorter employment histories than men or people not in a partnership (Kubicek et al. 2010; Lund and Villadsen 2005; Majeed et al. 2015; Wahrendorf 2015). In addition, high educational qualifications and advantaged economic circumstances were linked to later retirement (Komp et al. 2010). Secondly, health was linked to labour market participation, mostly because people in poor health claim disability pension (Harkonmäki et al. 2006; Leijten et al. 2015; Van Den Berg et al. 2010; van Rijn et al. 2014; Virtanen et al. 2014). This concerns a variety of chronic diseases, as well as mental and physical health functioning. A third important factor related to extended working lives refers t0 the workplace itself including work and employment conditions (van Solinge and Henkens 2014). For example, working in disadvantaged occupations (in particular elementary manual occupations with high physical demands) was linked to earlier retirement (Lain and Vickerstaff 2014), and bridge employment (Dingemans et al. 2016). Studies have also shown that self-employed people tend to have an extended working life (McNair et al. 2004), and that people reduce working time when they age (Nikolova and Graham 2014). Additionally, there is growing evidence that psychosocial working conditions are linked to early retirement. Specifically, evidence exists for high psychosocial demands, low control at work and low reward (Blekesaune and Solem 2005; Canivet et al. 2012; Carr et al. 2015; de Wind et al. 2014; Elovainio et al. 2005; Hintsa et al. 2015; Juvani et al. 2014; Laine et al. 2009; Robroek et al. 2013; Schnalzenberger et al. 2014; Siegrist and Wahrendorf 2011; Van Den Berg et al. 2010). Yet, in each of these studies psychosocial working conditions refer to midlife, and thus, conditions of older workers remain less explored.

In summary, the current study has two objectives: (a) a description of men and women working beyond state pension age, including their health, employment and working conditions, and (b) the comparison of health, employment and working conditions with retired in the same age group.

## Methods

### Data Source

We use data from the Survey of Health, Ageing and Retirement in Europe (SHARE, Release 5.0) collected between 2004 and 2012 across 16 European countries (Börsch-Supan et al. 2013b). SHARE is the first cross-national, longitudinal research project collecting data on a variety of social, economic and health-related topics among nationally representative samples of older adults. The survey started 2004–2005 in eleven countries (Sweden, Denmark, Germany, the Netherlands, Belgium, France, Switzerland, Austria, Italy, Spain and Greece), with on-going waves of data collection in two-year intervals. Two new countries joined SHARE in wave 2 (the Czech Republic and Poland) and four in wave 4 (Estonia, Hungary, Portugal and Slovenia), while Greece left SHARE at this point. In contrast to remaining waves, wave 3 of SHARE consists of a separate retrospective assessment of respondents’ previous life, with limited information on current information only (also called SHARELIFE). Although more recent data (wave 5, 2012–2013) of SHARE exists, we use wave 4 data (collected 2010–2012) and earlier, because information on current working conditions was not collected in wave 5 (only among new cohort members and newly employed people).

In each country, the initial sample is based on probability household samples where people above 50 years were interviewed plus their (possibly younger) partners in the household using Computer Assisted Personal Interviews (CAPI). At wave 1 the average household response rate was 62 % (with rates above 50 % in 8 out of 11 countries), 61 % in countries that joined SHARE in wave 2 and 56 % in wave 4. This is above average compared with other European surveys, and it corresponds to the observed general decline in survey participation over the past years both in Europe and worldwide. Notably, the sample size of SHARE increased substantially in wave 4, both because four new countries entered SHARE for the first time and because a large refreshment sample was drawn in countries that were already part of the survey. This not only includes respondents from younger cohorts (so called “refreshers” to maintain population representation), but also a new sample across the full age range, in order to increase the sample size (Malter and Börsch-Supan 2013). In terms of panel attrition, the average percentage of respondents lost between the waves was below 20. More details about SHARE and its methods are available online (www.share-project.org) or described elsewhere (Börsch-Supan et al. 2013a; Malter and Börsch-Supan 2013).

### Respondents

We restrict the sample to older men and women aged 65 years or older. This includes working people who answered that “employed or self-employed” best describes their current job situation and those who declared themselves “retired”. Then, we exclude people above the age of 80 because they may have particular work situations (e.g. working in a family run business). This results in a total sample of 8751 women and 8874 men (*n* = 17,625).

### Measures

*Sociodemographic variables:* In addition to sex and age, we include wealth, education and partnership situation. Wealth is based on household total net worth, both including financial wealth (savings, net stock value, mutual funds and bonds) and housing wealth (value of primary residence, other real estates, own business share and cars). For the analyses, we adjusted for household size in accordance with the OECD equivalent-scale, and thereafter calculated country-specific quartiles for the final sample (very low, low, high, very high). Because our wealth measure is based on accumulated savings and not on direct income, it may be more appropriate for older populations as an indicator of financial circumstances. Education is measured according to the International Standard Classification of Educational Degrees (ISCED-97) that we regroup into “low education” (pre-primary, primary or lower secondary education), “medium education” (secondary or post-secondary education), and “high education” (first and second stage of tertiary education). In the case of partnership, we use a binary indicator of whether the respondents lives with a partner or not (regardless the marital status).

*Work and employment conditions:* In sum, we use five measures to investigate work and employment conditions. In addition to employment status, occupational position and years in job, these are working hours and psychosocial work stress. For those who are employed, conditions refer to the current job. For those who are retired, we use information on the last job before entering retirement. In case persons participated for the first time in wave 4, this information was collected retrospectively. In the cases where people participated and worked in previous waves, this information is recovered from previous waves.

- *Employment status:* We distinguish between “employers” (self-employed with employees), “self-employed” who work on their own account (self-employed without employees), and salaried “employees” who are employed by an individual or an organisation. This corresponds to the primary classification scheme of the European Socio-economic Classification (ESeC) (Rose and Harrison 2007).
- *Occupational position:* As an indicator of occupational position, we regroup jobs into three categories based on the 10 major groups of International Standard Classification of Occupations (ISCO-88), developed by the International Labour Office (ILO 1990): “Managers and professionals” (groups 1 & 2, e.g. Director, chief executive, or health professional) “Other skilled workers” (major groups 3–8 & 10, e.g. medical assistant, cook or crane operator) and “Elementary occupations” (major group 9, e.g. cleaner, watchman or street vendor).
- *Years in job:* To assess years in job, we calculate how long the respondent has already worked in his or her job (for those employed), or the number of years spent in the last job (for those retired).
- *Working hours:* We use the self-reported total hours worked per week. For retired people, however, a retrospective assessment of working hours in the last job was not conducted in the survey (possibly due to recall bias). Information, therefore, could only be recovered for those retired people who participated and worked during previous waves, which results in a smaller subsample of retired people with information on working hours.
- *Psychosocial work stress:* We use two measures of psychosocial work stress, that rely on the original questionnaires of two prominent theoretical models of work stress: the demand-control (Karasek and Theorell 1990), and the effort-reward imbalance model (Siegrist et al. 2004). The former model identifies stressful work in terms of high demands in combination with low control, whereas the latter model measures stressful environment in terms of an imbalance between high efforts spent at work and low rewards received in turn. Both work stress models cover different, but equally relevant, aspects of the workplace, where lack of control and reward frustration matter most. The restrictions of a multi-disciplinary approach, though, meant it was not possible to include the full questionnaires of the two models. Therefore, SHARE includes abbreviated versions of original scales, where items that cover the core dimensions of the two models were selected on the basis of psychometric properties. For the demand-control model, the measurement was restricted to the control dimension as a core dimension that showed the highest predictive power (e.g. Marmot et al. 1997). This was measured by the sum score of two Likert-scale items, ranging from 2 to 8, with higher scores indicating lower control at work. To measure effort-reward imbalance, we use 2 out of 6 items to measure ‘effort’ and 5 out of 11 items to assess ‘reward’ at work. ‘Effort-reward imbalance’ was then calculated by dividing the sum score of the ‘effort’ items (nominator) though the sum score of the ‘reward’ items (adjusted for number of items; denominator). This results in a sum score ranging from 0.25 to 4 where, again, higher values are related to higher levels of work stress. This procedure is in line with previous studies, where both measures have been associated with mental health (Siegrist et al. 2012) or early retirement (Hintsa et al. 2015). As in the case of working hours, information on work stress was not assessed retrospectively among retired people, thus, leading to a smaller subsample of retired with available information. All single items and the exact wording are available as supplemantary material online.

*Health and Well-being Measures:* We include six binary indicators of poor health, all related to different aspects of health and well-being: First, we use poor self-perceived health (less than good), as assessed by a single question with five possible categories ranging from excellent to poor. Second, increased depressive symptoms are measured by the EURO-D depression scale. This scale includes 12-items for measuring depressive symptomatology and we defined more than 3 symptoms as increased levels. This was shown to be a valid and consistent indicator of elevated levels of depressive symptoms in cross-European studies (Prince et al. 1999). Third, we measure poor quality of life by the CASP-12v.1 questionnaire, a short version of the CASP-19 questionnaire (Hyde et al. 2015). An important characteristic of this instrument is that it neither focuses on respondents’ self-evaluation of quality of life, nor does it measure quality of life using measures of physical health as proxies. Rather, following existing literature of ageing (Giddens 1991; Laslett 1996), it identifies four domains and evaluates to what extent these are satisfied (using three 4-point Likert items for each domain). The four domains are: control (C), autonomy (A), self-realization (S) and pleasure (P). For the analyses, we created a sum-score with higher values indicting better quality of life and people scoring in the lowest quartiles (calculated for each country separately) were supposed to have “poor quality of life”. Details on psychometric properties of CASP-19 (and on its conceptual basis) are described elsewhere (Wiggins et al. 2008). Fourth, as a measure of functional limitations, we measured increased limitations in mobility (Wahrendorf et al. 2013). These limitations are based on a list of 10 difficulties in mobility, arm functions and fine-tuned motor function and two or more are used as an indicator of increased mobility limitations. Fifth, we combined results of the so-called “Wordlist-test” to measure low cognitive function (Adam et al. 2013). Hereby, respondents have to memorize and report a list with ten words twice in the frame of the interview (once immediate after reading the list and once a few minutes later). On this basis, we calculated a score counting the number of words recalled by the respondent (score ranging from 0 to 20). For reason of comparisons with the remaining health indicators, we again created a binary indicator for people scoring in the lowest quartiles (calculated for each country separately) to indicate “low cognitive functioning”. Finally, we included a measure of hand grip strength. Hand grip strength was measured twice on each hand using a dynamometer. For the analyses, we rely on the maximum grip strength measurement of the dominant hand, and sex-specific cut points are used to indicate poor strength (below 37 kg for men and below 21 kg for women (Sallinen et al. 2010).

### Analytical Strategy

**Fig. 1:**
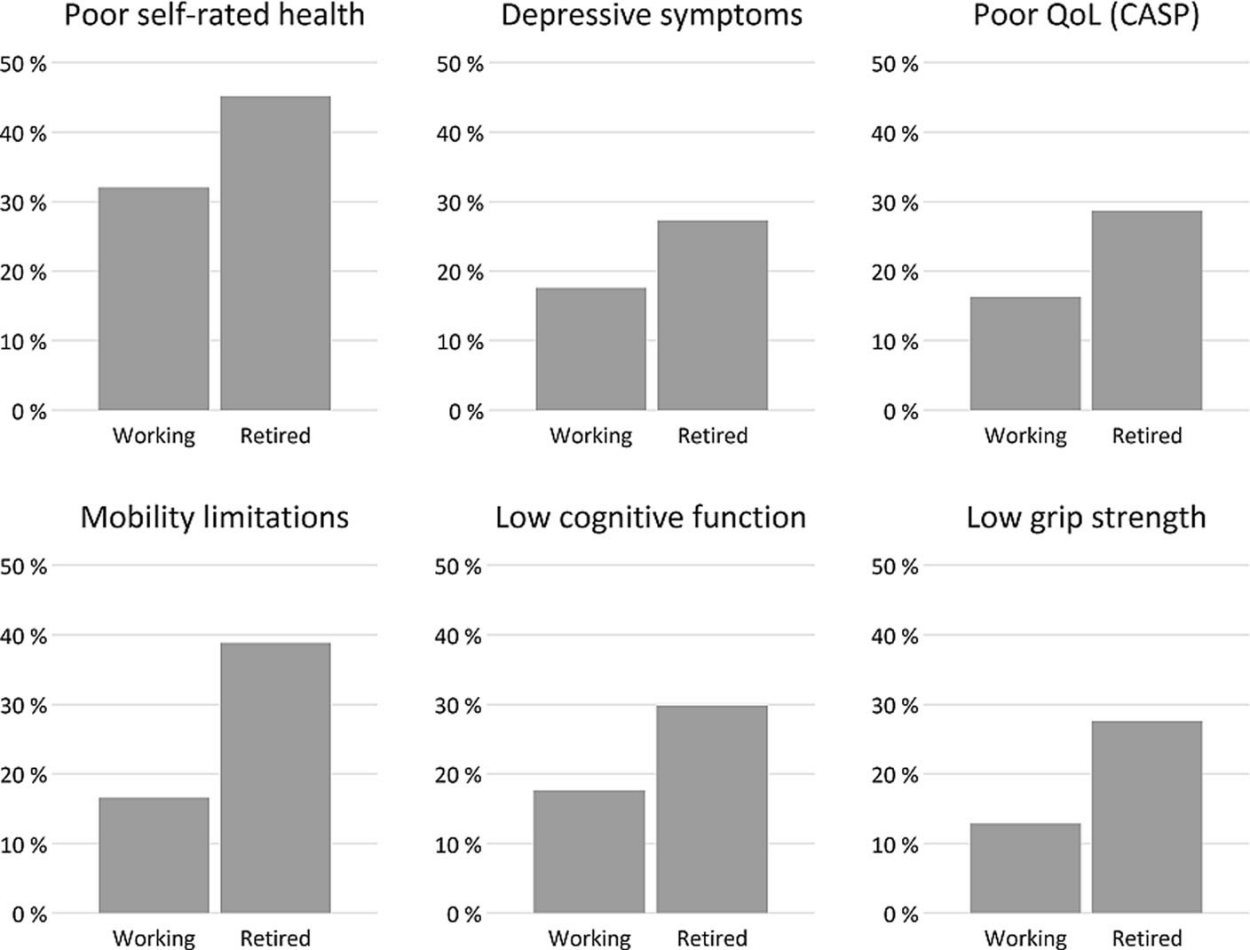
Prevalence of poor health by labour market situation among older men and women (aged 65 to 80 years) in percentage (n = 17,625)

To begin with, Table 1 gives a simple descriptive overview of sociodemographic and work-related measures, and additionally, we present their distribution in the two groups being studied, including tests of significance (*p*-values based on chi-square or t-test). Similarly, Table 2 presents an overview of poor health by employment situation that is summarised in Fig. 1.

**Table 1:** Distribution of sociodemographic characteristics and employment conditions for men and women aged 65 to 80 by labour market status: Observations (No.) and percentage (Col. %) or mean and standard deviation (SD)

	Working (n = 755)		Retired (n = 16,870)		Total (n = 17,625)	
	No.	Col % or mean (SD)	No.	Col % or mean (SD)	No.	Col % or mean (SD)
Sex						
Male	426	56.4	8448	50.1	8874	50.3
Female	329	43.6	8422	49.9	8751	49.7
p < 0.001						
Age						
Range: 65–80 years	755	68.44 (3.58)	16,870	71.54 (4.43)	17,625	71.41 (4.44)
p < 0.001						
Partnership						
Living with partner	546	72.3	12,063	71.5	12,609	71.5
Living as single	209	27.7	4807	28.5	5016	28.5
*p* = 0.628						
Education						
Low education	176	23.3	7458	44.2	7634	43.3
Medium education	286	37.9	6220	36.9	6506	36.9
High education	293	38.8	3192	18.9	3485	19.8
p < 0.001						
Wealth						
Very low wealth	101	13.4	4314	25.6	4415	25.1
Low wealth	138	18.3	4272	25.3	4410	25.0
High wealth	173	22.9	4230	25.1	4403	25.0
Very high wealth	343	45.4	4054	24.0	4397	24.9
p < 0.001						
Employment status						
Employed	498	66.0	14,836	87.9	1346	7.6
Self-employed	147	19.4	1199	7.1	15,334	87
Employer	110	14.6	835	4.9	945	5.4
p < 0.001						
Occupational position						
Managers and professionals	217	28.7	2958	17.5	3175	18
Other skilled workers	446	59.1	11,451	67.9	11,897	67.5
Elementary occupations	92	12.2	2461	14.6	2553	14.5
p < 0.001						
Working hours						
Range: 0–100 h	755	33.37 (16.32)	1085	35.38 (15.33)	1840	34.55 (15.77)
p < 0.001						
Years in job						
Range: 0–73 years	755	19.52 (14.91)	16,870	23.60 (13.86)	17,625	23.43 (13.93)
p < 0.001						
Low control						
Range: 2–8	754	4.00 (1.38)	1085	4.03 (1.41)	1839	4.02 (1.40)
p = 0.3039						
Effort-reward Imbalance						
Range: 0.25–4.0	736	0.82 (0.37)	1054	0.92 (0.42)	1790	0.88 (0.40)
p < 0.001						

Note. For those retired, working conditions refer to last job before retiring

**Table 2:** Distribution of poor health for men and women aged 65 to 80 by labour market status: Observations (No.) and percentage (Col. %) or mean and standard deviation (SD), (n = 17,625)

Categories	Working (n = 755)		Retired (n = 16,870)		Total (n = 17,625)	
	No.	Col %	No.	Col %	No.	Col %
Poor self-rated health						
No	513	67.9	9261	54.9	9774	55.5
Yes	242	32.1	7609	45.1	7851	44.5
p < 0.001						
Depressive symptoms						
No	622	82.4	12,257	72.7	12,879	73.1
Yes	133	17.6	4613	27.3	4746	26.9
p < 0.001						
Poor QoL (CASP)						
No	632	83.7	12,023	71.3	12,655	71.8
Yes	123	16.3	4847	28.7	4970	28.2
p < 0.001						
Mobility limitations						
No	630	83.4	10,309	61.1	10,939	62.1
Yes	125	16.6	6561	38.9	6686	37.9
p < 0.001						
Low cognitive function						
No	622	82.4	11,834	70.1	12,456	70.7
Yes	133	17.6	5036	29.9	5169	29.3
p < 0.001						
Low grip strength						
No	658	87.2	12,205	72.3	12,863	73.0
Yes	97	12.8	4665	27.7	4762	27.0
p < 0.001

Next, we estimate a serious of multivariable logistic regression models with random intercept and predict the likelihood of working with individuals (level 1) nested in countries (level 2) (Skrondal and Rabe-Hesketh 2010). This procedure allows for accurate adjustment for country affiliation, because the constant is allowed to vary across countries. This is important for our analyses, because we can focus on individual predictors, while accounting for country-specifics. Multilevel models were also compared to conventional logistic regression models (with country dummies), and revealed better model fits in all cases.

In sum, we present estimates of two types of regression models, calculated for each work-related and health-related measure (Tables 2 & 3). Model 1 presents estimates that are adjusted for sex, partnership situation, education and age of the respondent (linear) and model 2 additionally adjusts for education and wealth. Following recommendations of Mood (Mood 2010), we not only present odds ratios (OR) and their levels of significance, but also show the average marginal effects (AME) (Williams 2012). These are more intuitive and easier to interpret than OR, and also, they can be compared across different models (Mood 2010). More specifically, to obtain the AME for occupational position, for example, the predicted probabilities of being employed are computed for each occupational group separately (based on the logistic regression model). The differences in the probabilities then form the AME. For example, in a case where the AME for “managers and professionals” is 0.2 (with “elementary occupation” as the reference category), this means that on average the probability of working is 20 percentage points higher for “managers and professionals” than it is for the group of elementary occupations. In case of a continuous variable, the AME indicates the differences in the predicted probability of a one unit increase (e.g. one additional working hour). All calculations and graphs are produced with Stata 14.

**Table 3:** Association between working conditions and labour market participation among men and women aged 65 to 80 years: Adjusted odds ratios (OR) of working with level of significance, confidence intervals (CI 95 %) and average marginal effects [AME]

	Model 1			Model 2		
	OR	(CI 95 % )	[AME]	OR	(CI 95 % )	[AME]
Employment status						
Employed (ref.)	-			-		
Self-employed	8.09***	(6.39–10.23)	[0.107]	7.89***	(6.20–10.05)	[0.100]
Employer	7.00***	(5.41–9.07)	[0.094]	6.61***	(5.07–8.61)	[0.086]
Occupational position						
Managers and professionals	2.08***	(1.58–2.74)	[0.028]	0.93	(0.68–1.27)	[−0.003]
Other skilled workers	1.06	(0.83–1.36)	[0.002]	0.80	(0.62–1.03)	[−0.008]
Elementary occupations (ref.)	-			-		
Working hours	0.99	(0.99–1.00)	[−0.001]	0.99*	(0.98–1.00)	[−0.001]
Years in job	1.00	(0.99–1.00)	[−0.000]	1.00	(0.99–1.00)	[−0.000]
Low control	0.83***	(0.76–0.91)	[−0.025]	0.87**	(0.79–0.95)	[−0.019]
Effort-reward Imbalance	0.36***	(0.25–0.51)	[−0.133]	0.41***	(0.28–0.59)	[−0.115]

All estimates of Model 1 are adjusted for sex, age and partnership. Model 2 additionally adjusts for education and current wealth. For those retired, working conditions refer to the last job before retiring

* p < 0.05; ** p < 0.01; *** p < 0.001

## Results

### Sample Description

The total sample includes slightly more men than women (8874 vs. 8751), with a mean age of 71 years (see Table 1, last column for details). The large majority lives in a partnership. The average number of observations across countries is 1101, with the smallest number in Poland (523) and largest number in Estonia (2392) (not shown in the tables). Among all participants, 755 are working (4.3 %).

### Distribution of Variables by Labour Market Participation

In Table 1 we also see that people who are working between the ages of 65 and 80 years (first column) are more likely to be male, are slightly younger, have higher levels of education, and are wealthier than those who are retired (middle column). In addition, turning to work-related measures, the proportion of people who are self-employed is clearly higher in the group of workers than among those who are retired, as well as that people tend to work in advantageous occupational positions. Average working hours are somewhat lower among those working than the working hours retired people had in their last job. Turning to psychosocial working conditions, we see that levels of work stress were higher among retired people than the levels are among those who are working, particularly in the case of an effort-reward imbalance. In other words, it seems that levels of work stress are comparatively good for those working beyond state pension age. Finally, turning to the six indicators of health in Table 2, we see that health is worse among retired people than among older people working. The later finding is summarised in Fig. 1. All reported associations are statistically significant with *p*-values below 0.001.

### Multivariable Findings

Results of the multivariable regressions for work and employment conditions are presented in Table 3, and for health in Table 4. More specifically, for each condition we present adjusted odds ratios and AME of working between age 65 and 80. Estimates of model 1 are adjusted for gender, age and partnership, and model 2 additionally adjusts for education and wealth. By and large, findings confirm the descriptive results, with three findings worth noting:

**Table 4:** Association between health and labour market participation among men and women aged 65 to 80 years: Adjusted odds ratios (OR) of working with level of significance, confidence intervals (CI 95 %) and average marginal effects [AME]

	Model 1			Model 2		
	OR	(CI 95 % )	[AME]	OR	(CI 95 % )	[AME]
Poor self-rated health						
No (ref.)	-			-		
Yes	0.41***	(0.34–0.49)	[−0.027]	0.50***	(0.41–0.60)	[−0.021]
Depressive symptoms						
No (ref.)	-			-		
Yes	0.55***	(0.45–0.68)	[−0.017]	0.62***	(0.50–0.76)	[−0.014]
Poor QoL (CASP)						
No (ref.)	-			-		
Yes	0.55***	(0.45–0.67)	[−0.018]	0.65***	(0.53–0.80)	[−0.013]
Mobility limitations						
No (ref.)	-			-		
Yes	0.38***	(0.31–0.47)	[−0.027]	0.45***	(0.36–0.55)	[−0.022]
Low cognitive function						
No (ref.)	-			-		
Yes	0.63***	(0.52–0.77)	[−0.014]	0.81*	(0.66–1.00)	[−0.007]
Low grip strength						
No (ref.)	-			-		
Yes	0.57***	(0.46–0.72)	[−0.016]	0.65***	(0.52–0.82)	[−0.012]

All estimates of Model 1 are adjusted for sex, age and partnership. Model 2 additionally adjusts for education and current wealth

* p < 0.05; ** p < 0.01; *** p < 0.001

First, we see that people working between age 65 and 80 are more likely to be an employer or self-employed, to work as manager or professional, and under more favourable psychosocial working conditions. In detail, in model 1 the odds of working self-employed beyond state pension age are 8.09 times higher than in the last job of those who are retired. In terms of average marginal effects, this is a difference of 10.7 percentage points between the two groups (AME = −0.107). Secondly, it is worth noting that the estimates for occupational position are attenuated, once education and wealth are included in model 2, possibly because people with high education and high wealth are both more likely to work in higher-skilled jobs and to work between age 65 and 80. Finally, turning to Table 4, the state of health is consistently worse among older retired respondents than among workers, even after adjusting for sex, age, partnership, education and wealth.

## Discussion

In this study we describe the working and employment conditions of men and women working between 65 and 80 years of age across Europe, and contrast them with previous conditions of retired people in the same age group. Three major findings result from our analyses: First, we observe that about one in three people working between 65 and 80 is self-employed (either with or without employees) or works in an advantageous occupational position as a manager or professional. When comparing each of these conditions with those which retired people had in their last job, proportions are lower. This supports the assumption that those with extended working lives are more likely to be self-employed or to work in higher-skilled employments. Maybe self-employed workers are less likely to have a generous pension, resulting in increased incentives (and necessity) to work longer. And high-skilled workers may continue working because they enjoy it, even if they may expect a generous pension. This is further supported by the second main finding showing that psychosocial working conditions are generally better among those working beyond state pension age. Third, with regard to health, we find that physical and mental health tends to be better among those working compared with retired people.

Overall, these findings are concordant with current research, specifically studies which investigate employment and working conditions in conjunction with retirement (Carr et al. 2015; Hintsa et al. 2015; Juvani et al. 2014; Robroek et al. 2013; Van Den Berg et al. 2010). Yet, by using information on the current circumstances of men and women working beyond state pension age, we add to this literature and elucidate working conditions of extended working lives more explicitly, because the used measures directly refer to current conditions and not to conditions at an earlier stage of the working career. Furthermore, a more comprehensive comparison than in previous occupational cohort studies was possible because all retired persons were included in our study, and not only those who retired between baseline and follow-up, as was the case in occupational cohort studies. Our study thus adds to studies focussing on the relationship between psychosocial working conditions and retirement(Blekesaune and Solem 2005; Carr et al. 2015; Elovainio et al. 2005; Hintsa et al. 2015; Juvani et al. 2014; Schnalzenberger et al. 2014).

While our study profits from several strengths (large study sample, high quality standards of data collection and comprehensive assessment of employment and working conditions based on validated questionnaires), we have to consider several limitations. First, we have to bear in mind that response rates were not very high in some countries and that attrition could have led to a selective sample in our study, because some groups are more likely to participate in the survey than others. For example, attrition and non-response are likely to be higher among more disadvantaged groups, thus leading to a general under-report of disadvantaged jobs in our study. The question, however, is to what extent this also affects our comparison, because the described selection may both apply to working and retired persons. Or, in case the described selection is higher among retired people, we may have even under-estimated the size of the difference between both groups. Response rates, however, were above European average and analyses comparing the SHARE sample to other prominent European surveys (e.g. the European Social Survey) confirmed that the sample represents the working population quite well (Börsch-Supan and Mariuzzo 2005). A second limitation refers to additional variables that we may have included into our analyses, for example, engagement in voluntary work or caregiving activities beyond paid employment. Future analyses may address this aspect in more detail, in particular by studying circumstances under which people are more likely to combine both paid and non-paid work in older ages. Previous studies, for example, indicate that people are more likely to combine paid work and volunteering at later stages of their working life, if they are able to remain working while reducing working hours (Erlinghagen 2010; Sugihara et al. 2008). Third, we have to bear in mind that information on work stress and on working hours could only be recovered for the subsample of retired respondents that worked at previous waves, because this information was not assessed retrospectively. It is, therefore, likely that we have underestimated the differences in work stress between those who work beyond the state pension age and those who are retired in the same age group, possibly since workers with higher levels of work stress may have left the workforce at younger ages before study onset. Fourth, although our multilevel analyses did consider country variations of the employment and working conditions studied, we may nevertheless ask if results apply for every country, specifically, if better employment and working conditions for workers beyond age 65 years can be found in each country under study. For example, existing pension practices and resulting financial resources of older people do vary across Europe which lead to country-specific motivations of working beyond state pension age. Still, our study clearly show that a large majority of the variations of working trends is attributed to individual characteristics, highlighting the importance of individual predictors in explaining employment among workers beyond age 65 years. In a similar vein, we may also ask if results differ between men and women. Yet, to investigate these questions in more detail, we clearly need larger sample sizes allowing meaningful analyses of subgroups, particular for the small group of people with extended working lives (by country, sex or both). Finally, we have to ask how our results align with rapidly changing workforces in Europe (Eurofound 2015). In fact, although we covered a range of European countries, our data on working conditions of workers aged between 65 and 80 years was collected in 2010–2012 (wave 4). Newest SHARE data (wave 5), however, do not collect information on work stress (only among new cohort members and newly employed people). Therefore, future studies may test if our results can be replicated with more recent data.

In conclusion, the findings deliver empirical evidence that extended working lives are more common under specific employment circumstances (self-employed, advantaged occupational positions) and under favourable psychosocial circumstances (high control and balance between effort and reward), and additionally, that people working beyond the age of 65 years are in considerably good mental and physical health. This underlines that not every person will end up in an extended working life and shows that policies aiming at increasing the state pension age beyond the age of 65 years put pressure on specific groups of men and women.

## Electronic supplementary material

ESM 1 (DOCX 14 kb)
