# Supplementary material for: Who in Europe Works beyond the State Pension Age and under which Conditions? Results from SHARE
Source: J Popul Ageing. 2016 Sep 23;10(3):269–85. doi: 10.1007/s12062-016-9160-4 (PMC5569122; doi:10.1007/s12062-016-9160-4)
Supplement: Supplementary file 1 — (DOCX 14 kb) [file 12062_2016_9160_MOESM1_ESM.docx]

**Supplementary tables**

Table S1. Items measuring stressful working conditions

| **Dimension** | **Item** |
| --- | --- |
| Control | 1. I have very little freedom to decide how I do my work. 2. I have an opportunity to develop new skills. |
| Effort | 1. My job is physically demanding. 2. I am under constant time pressure due to a heavy workload. |
| Reward | 1. I receive adequate support in difficult situations. 2. I receive the recognition I deserve for my work. 3. Considering all my efforts and achievements, my salary is adequate 4. My job promotion prospects are poor. 5. My job security is poor. |

Note. The interviewers' spoken text is as follows: "I am now going to read some statements people might use to describe their work. We would like to know if you feel like this about your present job. Please tell me whether you strongly agree, agree, disagree or strongly disagree with each statement."
